# Differential responses of arbuscular mycorrhizal fungal communities to mineral and organic fertilization

**DOI:** 10.1002/mbo3.920

**Published:** 2019-08-08

**Authors:** Jia Liu, Jie Zhang, Daming Li, Changxu Xu, Xingjia Xiang

**Affiliations:** ^1^ Soil and Fertilizer & Resources and Environment Institute Jiangxi Academy of Agricultural Sciences Nanchang China; ^2^ College of Water Conservancy and Ecological Engineering Nanchang Institute of Technology Nanchang China; ^3^ Jiangxi Institute of Red Soil Jinxian China; ^4^ Anhui Province Key Laboratory of Wetland Ecological Protection and Restoration, School of Resources and Environmental Engineering Anhui University Hefei China

**Keywords:** agricultural ecosystem, arbuscular mycorrhizal fungi, feedback, fertilization, sequencing

## Abstract

Agricultural fertilization is used extensively to increase soil fertility and maximize crop yield. Despite numerous studies on how fertilization influences plant and bacterial communities, little is known about the roles of long‐term application of different fertilizers in shaping arbuscular mycorrhizal fungal (AMF) community structures in a comparative manner. The response of AMF community to 28 years of chemical and organic fertilization was investigated using the Illumina Mi‐Seq platform. Soil AMF community composition showed significant and differential responses to long‐term fertilization. Changes in available phosphorus (AP) content were the primary driver shaping AMF community composition. Chemical fertilization significantly decreased AMF alpha‐diversity, whereas the alpha‐diversity remained equally high in organic fertilization treatment as in the control. In addition, soil AMF alpha‐diversity was negatively and positively correlated with elevated soil nutrient level following chemical and organic fertilization, respectively. Plants could directly acquire sufficient nutrients without their AMF partners after chemical fertilization, while plants might rely on AMF to facilitate the transformation of organic matter following organic fertilization, indicating that chemical fertilization might reduce the reliance of plants on AMF symbioses while organic fertilization strengthened the symbiotic relationship between plants and their AMF partners in agricultural ecosystems. This study demonstrated that AMF communities responded differently to long‐term chemical and organic fertilization, indicating that organic fertilization might activate belowground AMF function to maintain soil nutrients and benefit the sustainable development of agriculture.

## Graphical abstract

Soil AMF community composition showed significant response to long‐term fertilization whose effects were different following chemical and organic fertilizer treatments. Besides, soil AMF community composition was shaped by soil nutrient contents which were asymmetrically changed following chemical and organic fertilization, suggesting that the different responses of AMF community composition were mediated by fertilization‐type‐induced shifts in soil nutrient contents. Chemical fertilization significantly decreased AMF alpha‐diversity which showed similar level following organic fertilization relative to control samples. In addition, soil AMF alpha‐diversity showed negative and positive correlation with elevated soil nutrient level following chemical and organic fertilization, respectively. Plant could directly acquire sufficient nutrients without their AMF partner after chemical fertilization, while plant might rely on AMF to facilitate the transformation process of organic matters following organic fertilization, indicating that chemical fertilization might reduce reliance of plants on AMF symbioses while organic fertilization strengthened the symbiotic relationship between plant and AMF partner in agricultural ecosystem.

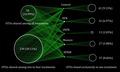

## 1 INTRODUCTION

Chemical fertilizers have been extensively applied in agricultural ecosystems to increase soil fertility and maximize yield (Mozumder & Berrens, 2007; Savci, 2012; Sun, Zhang, Guo, Wang, & Chu, 2015). Previous studies have demonstrated that balanced chemical fertilization (NPK) improved crop yield relative to nutrient‐deficient fertilization (Lin et al., 2012; Zheng et al., 2009). Nitrogen (N) fertilization alone has a marked effect on soil phosphorus (P) availability, as N addition induces a shift in soil nutrient limitation from N to P and vice versa, which might be detrimental for plant growth (Lü et al., 2013). Further, soil microorganisms have more efficient metabolism under balanced fertilization than under nutrient‐deficient fertilization (Ge, Zhang, Zhang, Yang, & He, 2008; He, Zheng, Chen, He, & Zhang, 2008; Zheng et al., 2009). Soil microorganisms are strongly associated with soil fertility (Hart, DeLuca, Newman, MacKenzie, & Boyle, 2005; He et al., 2008). Balanced chemical (NPK) fertilization ameliorates soil properties, increases nutrient availability, and leads to high soil productivity (Lin et al., 2012). However, large amounts of chemical fertilizers may cause problems, such as soil degradation, environmental pollution, and reduction in soil biodiversity (Blanco‐Canqui & Schlegel, 2013; Byrnes, 1990; Mozumder & Berrens, 2007). Long‐term chemical fertilization significantly decreased soil organic carbon (SOC) content (Li et al., 2014; Mack, Schuur, Bret‐Harte, Shaver, & Chapin, 2004), which forms the basis of soil fertility and sustainable agriculture (Reeves, 1997). Soil acidification is related to long‐term chemical fertilization in agricultural ecosystems (Guo et al., 2010; Yue, Zhang, Shi, Yao, & Zhang, 2016). The efficiency of fertilization on yield decreases as the duration of chemical fertilization increases (Horrigan, Lawrence, & Walker, 2002). Long‐term chemical fertilization affects not only soil properties, but also belowground microbial parameters. Soil microbial biomass (Allison et al., 2013; Lovell, Jarvis, & Bardgett, 1995) and microbial diversity (Coolon, Jones, Todd, Blair, & Herman, 2013; Ramirez, Lauber, Knight, Bradford, & Fierer, 2010) showed significant decrease in response to long‐term chemical fertilization. Long‐term chemical fertilization triggered dramatic shifts in soil bacterial (Ramirez et al., 2010; Sun et al., 2015; Wakelin et al., 2007; Zhao et al., 2014) and fungal (Avio et al., 2013; He et al., 2016; Klabi et al., 2015) community composition.

Although chemical fertilization has detrimental effects on agricultural ecosystems, organic fertilizers are typically beneficial to the soil microbial community. Compared to chemical fertilizers, the release of nutrients from organic fertilizers is more sustainable (Chen, 2006; Shirani, Hajabbasi, Afyuni, & Hemmat, 2002), which increases product quality (Fliessbach, Oberholzer, Gunst, & Mäder, 2007; Langenkämper et al., 2006; Mäder et al., 2007). The application of organic fertilizer might improve hydraulic conductivity and decrease bulk density in agricultural ecosystems (Hati, Mandal, Misra, Ghosh, & Bandyopadhyay, 2006; Sun et al., 2015). Organic fertilization significantly increased SOC content which is one of the most important indicators of soil quality (Chan, 2008; Reeves, 1997). Organic fertilization enhanced soil fertility and biodiversity, which reduced dependence on external inputs, suggesting that organic fertilizer might provide a realistic alternative to conventional agricultural practices (Mäder, Edenhofer, Boller, Wiemken, & Niggli, 2000; Mäder et al., 2002). Organic fertilizers offer soil carbon resources and abundant substrates to improve microbial habitats (Miller et al., 2008; Philippot, Hallin, & Schloter, 2007) and increase bacterial diversity (Chaudhry, Rehman, Mishra, Chauhan, & Nautiyal, 2012; Sun et al., 2015). Previous studies have also shown that addition of organic matter significantly increased the population size of ammonia‐oxidizing bacteria (Wang, Zhu, Song, Wang, & Yin, 2014). Some studies argued that the release of nutrients from organic manure might be too slow for crop growth (Bandyopadhyay, Misra, Ghosh, & Hati, 2010), while the mixed application of organic and chemical fertilizers might be better for increasing soil fertility and maximizing crop yield relative to solo application of organic or chemical fertilizers (Aguilera, Motavalli, Gonzales, & Valdivia, 2012; Bokhtiar & Sakurai, 2005).

Many studies have demonstrated how long‐term fertilization affects biochemical properties and microbial communities, with limited information on the response of arbuscular mycorrhizal fungal (AMF) communities to long‐term application of chemical and organic fertilizers. Arbuscular mycorrhizal fungal could regulate organism interactions, provide nutrients, and promote stress tolerance for their hosts (Chu et al., 2016; Moora, Öpik, Sen, & Zobel, 2004; Xiang et al., 2016). However, the mutualistic symbioses would be changed under high‐nutrient conditions, which would allow plants to directly acquire enough nutrients (Lin et al., 2012), resulting in a decrease in soil AMF activity and/or diversity (Johnson, 2010; Xiang et al., 2016). Fertilization significantly increased soil nutrient level (Mozumder & Berrens, 2007; Sun et al., 2015), which might trigger significant shifts in AMF community composition and diversity. In addition, the release of nutrients differs between chemical and organic fertilization, which might result in different responses of AMF communities to these two fertilizer types. Understanding AMF communities following fertilization might be crucial for predicting how fertilization will affect agricultural ecosystems. We investigated the response of soil AMF communities to 28 years of chemical and organic fertilization legacy at an Agro‐Ecological Experimental Station. Our main hypotheses were that (a) AMF community composition would differentially responded to long‐term chemical and organic fertilization; (b) fertilization significantly decreased soil AMF diversity due to high soil nutrient availability after application of chemical and organic fertilizers.

## 2 MATERIALS AND METHODS

### 2.1 Site selection and soil sampling

The fertilization experiment, which began in 1986, is located in Jinxian County, Jiangxi Province (28°37′N, 116°26′E; 26 m; Yue et al., 2016). The soil is classified as Ultisol (Soil Survey Staff, 2010). The annual average precipitation and mean temperature are 1727 mm and 17.5°C, respectively. The field site is used to cultivate corn continuously. Corn is planted in early April and ripens in mid‐July. The plots were arranged in a randomized block design (size 5.55 m × 4.00 m = 22.2 m^2^). Our study included five treatments with three replicates: control (without fertilization); balanced chemical fertilization (NPK; 60 kg N ha^−1^ yr^−1^, 13 kg P ha^−1^ yr^−1^ and 33 kg K ha^−1^ yr^−1^); double dozes of NPK fertilization (2NPK; 120 kg N ha^−1^ yr^−1^, 26 kg P ha^−1^ yr^−1^ and 66 kg K ha^−1^ yr^−1^); organic fertilization (fresh pig manure at 15 t ha^−1^ yr^−1^); and NPK plus manure treatment (NPKM; fresh pig manure at 15 t ha^−1^ yr^−1^, 60 kg N ha^−1^ yr^−1^, 13 kg P ha^−1^ yr^−1^ and 33 kg K ha^−1^ yr^−1^). Urea, calcium magnesium phosphate, and potassium chloride were chosen for mineral N, P, and K fertilizers, respectively. The fresh pig manure contained 70.4% moisture. The dry manure contained 28.3 g N kg^−1^, 10.3 g P kg^−1^, and 9.8 g K kg^−1^. The manure and all P fertilizer were applied as basal fertilizers before soil plowing (10 days before planting corn). Half of the total N and K fertilizers were incorporated as basal fertilizers, and the other half applied at the V12 stage (i.e., the 12th leaf of the corn was fully expanded). Soils were sampled on 12 May 2014 (i.e., following continuous fertilization for 28 years). In each site, surface soils (0–10 cm) were collected from four corners of a plot (1.5 m from the plot edge) and then combined into one sample. The samples were immediately sieved to completely mix and transported to the laboratory with refrigeration within 24 hr. The samples were mixed thoroughly and then divided into two parts: One part was stored at 4°C for soil chemical analysis, and the other was stored at −20°C for DNA extraction.

### 2.2 Crop yield and soil property analyses

Ripe corn was collected and weighed to record the yield. Soil pH was measured after shaking a soil–water suspension (1:5 wt/vol) for 30 min. The classical methods were applied for measuring soil available phosphorus (AP; Ståhlberg, 1980), total phosphorus (TP; Bowman, 1988), SOC, and total nitrogen (TN; Walkley & Black, 1934). Soil dissolved organic carbon (DOC), total dissolved N (TDN), and mineral nitrogen were extracted by adding 50 ml of 0.5 M K_2_SO_4_ to 10 g fresh soil, shaking for 1 hr, and then vacuum filtering through glass fiber filters (Fisher G4, 1.2 μm pore space). Ammonium (NH_4_^+^) and nitrate (NO_3_^−^) contents in the extracts were determined colorimetrically by automated segmented flow analysis (Bran + Luebbe AAIII) using the salicylate/dichloroisocyanuric acid and cadmium column/sulfanilamide reduction methods, respectively. Dissolved organic carbon and TDN were determined using a TOC‐TN analyzer (Shimadzu). Dissolved organic nitrogen (DON) was calculated as follows: DON = TDN − NH_4_^+^ − NO_3_^−^.

### 2.3 Soil DNA extraction

DNA extractions were carried out on 0.5 g soil according to the manufacturer's instructions (FastDNA® SPIN Kit for soil, MP Biomedicals). The extracted DNA was dissolved in 50 μl TE buffer, quantified by NanoDrop ND‐1000 (Thermo Scientific) and stored at −20°C.

### 2.4 PCR and amplicon library preparation

PCR was carried out in 50 μl reaction mixtures containing each deoxynucleoside triphosphate at a concentration of 200 μM, forward or reverse primers at a concentration of 0.4 μM, 2 U of Taq DNA polymerase (TaKaRa), and 50 ng of DNA. The following cycling parameters were used as follows: 35 cycles (95°C for 45 s, 56°C for 45 s, and 72°C for 45 s) were performed with a final extension at 72°C for 10 min. Triplicate reaction mixtures per sample were pooled together and purified using an agarose gel DNA purification kit (TaKaRa), and quantified using NanoDrop ND‐1000 (Thermo Scientific, USA) ranging from 64.1 to 92.5 ng/μl. The bar‐coded PCR products were pooled in equimolar amounts (10 pg for each sample) before sequencing.

### 2.5 Processing of sequence data

The primer sets of AMV4.5NF/AMDGR were chosen to amplify the fragments of 18S rRNA gene (Lumini, Orgiazzi, Borriello, Bonfante, & Bianciotto, 2010) for the Mi‐Seq platform (PE 300) at Majorbio. The raw sequences were processed using QIIME software (Caporaso et al., 2010). Sequences with the quality score and length <25 and 200 bp, respectively, were removed. Sequences were grouped into operational taxonomic units (OTUs; 97% identity threshold) by UCLUST (Edgar, 2010). Singleton and chimera OTUs were deleted. The most abundant sequence within each OTU was chosen as the representative sequence which was checked against the MaarjAM AMF database (Öpik et al., 2010). The detailed parameters for bioinformatics were shown in the study of Xiang et al. (2016). We rarified the abundance matrix to 7,700 sequences (the lowest read depth across the samples) per sample to obtain normalized relative abundances.

### 2.6 Statistical analysis

One‐way ANOVA was conducted to analyze AMF alpha‐diversity and the relative abundance of AMF orders (normal distribution verified by Kolmogorov–Smirnov test; *p* > .05 in all cases). Pearson correlations were performed between soil AMF data (i.e., alpha‐diversity and the relative abundance of AMF orders) and chemical properties. The effects of fertilization on soil AMF community composition were investigated by nonmetric multidimensional scaling (NMDS) and analyses of similarities (ANOSIM). Mantel tests were conducted to identify factors that showed significant correlation with AMF community composition. The variance inflation factor (VIF) was used to examine multicollinearity of soil metadata whose values <3 were chosen (Zuur, Ieno, & Elphick, 2010) for variance partitioning analysis (VPA).

## 3 RESULTS

### 3.1 Soil chemical properties and crop yield

Chemical fertilizers significantly decreased soil pH, while manure (M) and manure with NPK (NPKM) additions significantly increased soil pH relative to the control (Table A1). Compared to the control, NPK addition significantly increased SOC and DON; 2NPK addition significantly increased soil TP, SOC, NH_4_^+^, AP, and DOC; and M and NPKM additions significantly increased soil TN, TP, SOC, NO_3_^−^, AP, DON, and DOC (Table A1). Corn yield significantly increased following fertilization, and the effect on yield was stronger when using organic manure than when using chemical fertilizer (Table A1). A previous study showed the corn yield from 1986 to 2008 in the same study area (Huang, Zhang, Yu, & Huang, 2010), which was consistent with the current study. In addition, fertilization led to significant increasing trends in corn yield for the M and NPKM treatments, while little change was observed following chemical fertilization on the same timescale (Huang et al., 2010).

### 3.2 Arbuscular mycorrhizal fungal alpha‐diversity

**Figure 1:**
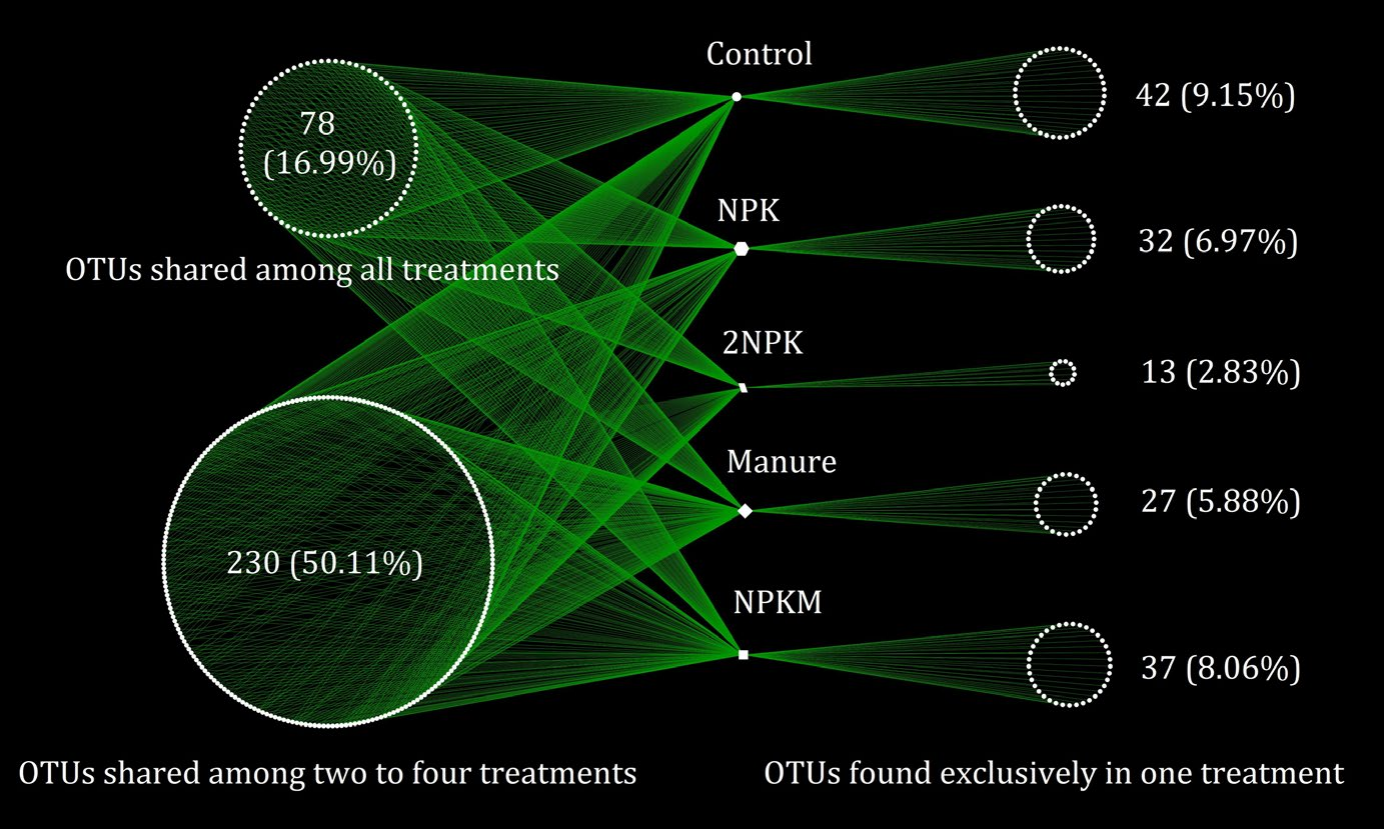
An OTU (operational taxonomic unit) network showing the interactions of the OTUs among all the samples from different treatments. Each point represents one independent AMF fungal OTU. Operational taxonomic units in the right column were unique to one treatment, whereas those in the left column belonged to multiple treatments

**Figure 2:**
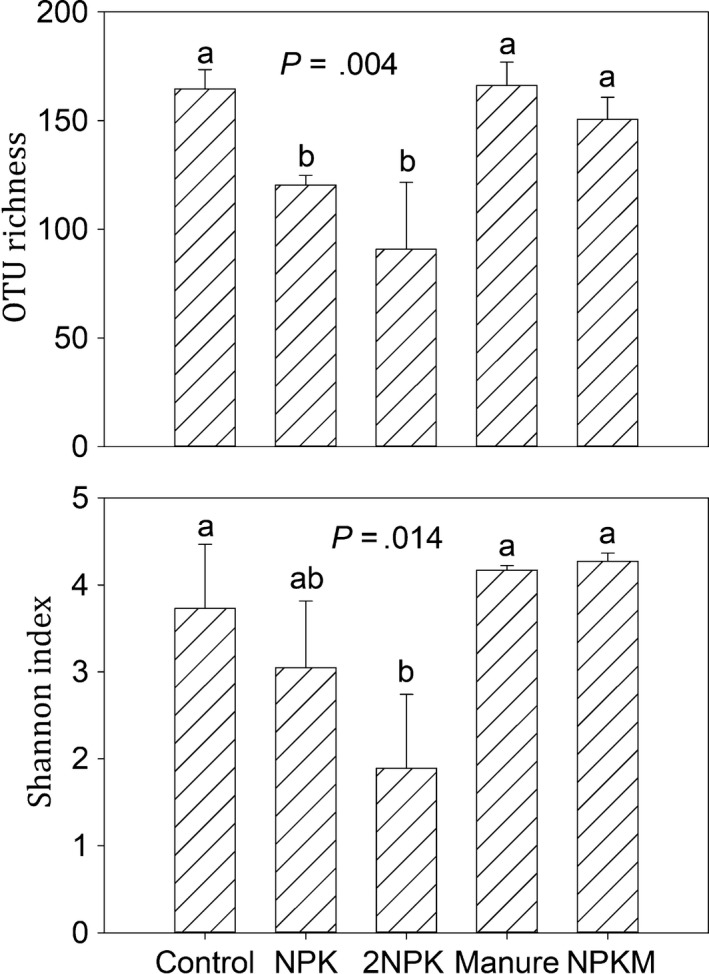
Soil AMF alpha‐diversity calculated at a rarefaction depth of 7,700 randomly selected sequences per sample in soils across different treatments. Different letters represent significant differences from Tukey's HSD comparisons (*p* < .05). Error bars denote standard deviation

A total of 180,528 quality AMF sequences and 459 distinct AMF OTUs were identified across all soil samples. There were 42, 32, 13, 27, and 37 unique AMF OTUs in the control, NPK, 2NPK, Manure, and NPKM treatments, respectively, and 16.99% of AMF OTUs (78) were found in all four treatments (Figure 1). Compared to the control, chemical fertilizer significantly decreased AMF OTU richness, and the high amount of NPK addition (2NPK) significantly decreased AMF Shannon index (Figure 2). However, AMF alpha‐diversity showed a similar level following M and NPKM additions relative to control soils (Figure 2). Operational taxonomic unit richness was significantly correlated with soil pH, and Shannon index was significantly correlated with pH, AP, and DON among all samples (*p* < .05 in all cases; Table 1). Arbuscular mycorrhizal fungal alpha‐diversity was significantly negatively correlated with soil nutrient contents (i.e., TP, AP, and SOC; *p* < .05 in all cases) and corn yield (*p* < .05 in both cases) in subset treatments of control, NPK, and 2NPK, while AMF alpha‐diversity was positively correlated with nutrient contents (i.e., TP, AP, SOC, DOC, and DON; *p* < .05 in all cases) and corn yield (*p* < .05 in both cases) in subset treatments of control, manure, and NPKM (Table 1). The results indicated that different fertilizer types might trigger differential feedback among soil nutrients, aboveground productivity, and belowground AMF alpha‐diversity.

**Table 1:** Pearson correlations (*r*) between AMF alpha‐diversity and variables (i.e., chemical properties and crop yield)

Variables	All samples		CK + NPK + 2NPK		CK + Manure + NPKM	
	OTU	Shannon	OTU	Shannon	OTU	Shannon
pH	**0.636** *	**0.684** **	**0.787** *	0.601	**0.852** **	**0.861** **
TN	0.249	0.389	**−0.765** *	−0.636	0.579	0.604
TP	0.301	0.484	**−0.754** *	**−0.662** *	**0.791** *	**0.871** **
NO_3_^−^	0.023	0.186	−0.504	−0.425	0.265	0.361
NH_4_^+^	−0.482	−0.365	−0.460	−0.279	−0.394	−0.470
AP	0.395	**0.554** *	**−0.714** *	**−0.684** *	**0.810** **	**0.887** **
SOC	0.108	0.319	**−0.687** *	**−0.705** *	**0.819** **	**0.892** **
DOC	0.289	0.506	−0.658	−0.384	**0.800** **	**0.902** **
DON	0.473	**0.633** *	−0.126	0.311	**0.829** **	**0.871** **
CY	−0.063	0.143	**−0.817** **	**−0.674** *	**0.694** *	**0.758** *

Abbreviations: AP, available phosphorus; CY, corn yield; DOC, dissolved organic carbon; DON, dissolved organic nitrogen; SOC, soil organic carbon; TN, total nitrogen; TP, total phosphorus.

Significant correlations are shown in bold (*p* < 0.05).

* *p* < .05.

** *p* < .01.

### 3.3 Arbuscular mycorrhizal fungal community composition

**Figure 3:**
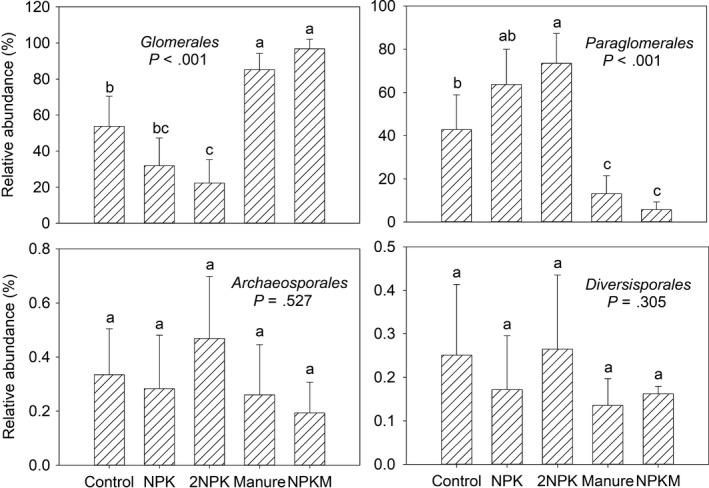
Relative abundances of dominant AMF orders across treatments. Error bars denote standard deviation; different letters represent significant differences from Tukey's HSD comparisons (*p* < .05)

**Figure 4:**
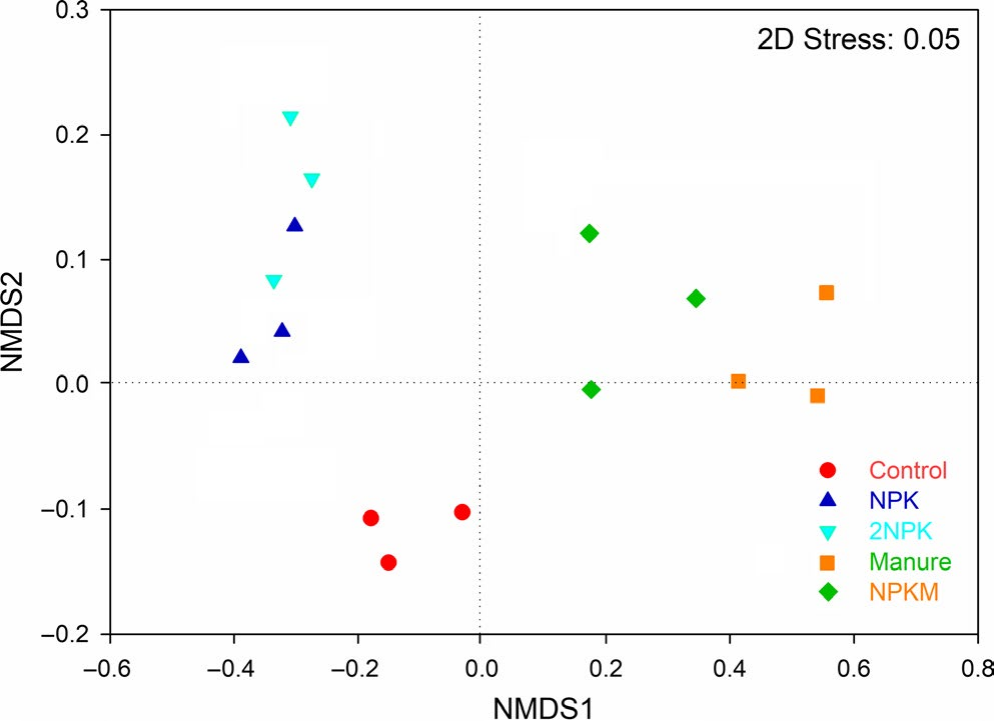
Nonmetric multidimensional scaling (NMDS) plot showing AMF community composition across treatments

The results showed that the dominant AMF orders were *Glomerales* (58.01%) and *Paraglomerales* (39.19%). In addition, *Archaeosporales* (0.29%) and *Diversisporales* (0.16%) were also found with low abundances in soils. Compared to the control, the relative abundances of *Glomerales* and *Paraglomerales* significantly decreased and increased in 2NPK treatment, respectively (Figure 3). There was greater relative abundance of soil *Glomerales* and lower relative abundance of soil *Paraglomerales* with application of M and MNPK in comparison with the control sample (Figure 3). Fertilization resulted in significant shifts in soil AMF community composition (Figure 4; Table A2). In addition, AMF community composition responded differentially between chemical and organic fertilizer additions (Figure 4; Table A2).

Soil AMF community composition was significantly correlated with pH, TN, TP, AP, SOC, DOC, DON, and corn yield (*p* < .05 in all cases; Table 2). Soil AP showed the highest correlation with AMF community composition (*p* = .001). In addition, VPA demonstrated that AP contributed to the largest proportion of AMF community variation (Table A3). The relative abundance of *Glomerales* was positively correlated with soil pH, TN, TP, AP, SOC, DOC, and DON (*p* < .05 in all cases; Table A4). Soil pH, TN, TP, AP, SOC, DOC, and DON were negatively correlated with the relative abundance of *Paraglomerales* (*p* < .05 in all cases; Table A4). Soil NO_3_^−^ and NH_4_^+^ contents were negatively related to the relative abundance of *Archaeosporales* (*p* < .05 in both cases; Table A4). The relative abundance of *Diversisporales* was negatively correlated with soil NO_3_^−^ (*p* < .05; Table A4).

**Table 2:** Correlation coefficients (*r*) and significance (*p*) were determined by Mantel tests: comparing differences between samples in AMF community composition to differences between samples in variables (i.e., soil properties and crop yield)

Variables	Mantel test	
	*r*	*p*
pH	**.659**	**.001**
TN	**.555**	**.004**
TP	**.736**	**.001**
NO_3_^−^	.096	.141
NH_4_^+^	.118	.835
AP	**.768**	**.001**
SOC	**.443**	**.005**
DOC	**.712**	**.001**
DON	**.706**	**.001**
CY	**.460**	**.003**

Abbreviations: AP, available phosphorus; CY, corn yield; DOC, dissolved organic carbon; DON, dissolved organic nitrogen; SOC, soil organic carbon; TN, total nitrogen; TP, total phosphorus.

## 4 DISCUSSION

Our first hypothesis was that soil AMF community composition would respond differentially to long‐term chemical and organic fertilization. In this study, long‐term fertilization led to significant changes in soil AMF community composition. Previous studies also demonstrated the significant response of AMF community to fertilization in grasslands (Chen et al., 2014; Liu et al., 2012; Xiang et al., 2016) and agricultural ecosystems (Antoninka, Reich, & Johnson, 2011; Avio et al., 2013), suggesting that the AMF community is sensitive to changes in soil nutrients (Johnson, 1993; Lin et al., 2012; Liu et al., 2012). In addition, differential responses in AMF community composition to fertilizer types were detected in this study, suggesting that fertilizer type might be an important factor in mediating the relationships between plants and their AMF partners. We found that soil AMF community composition was significantly correlated with soil nutrient contents (i.e., AP and SOC) which were significantly higher following organic fertilization than chemical fertilization, indicating that the differential responses of AMF community composition might be mediated by fertilization‐type‐induced changes in soil nutrient level (Xiang et al., 2016).

We also hypothesized that fertilization significantly decreased AMF diversity due to high soil nutrient availability following addition of chemical and organic fertilizers. Previous studies have demonstrated that plants might directly absorb enough nutrients (e.g., AP and nitrogen) without help from AMF partners following chemical fertilization, and then, plants decrease their dependence on belowground AMF symbioses, which increases competition among AMF taxa to form symbioses with plants (Johnson, 1993; Lin et al., 2012). Moreover, plants decrease their belowground allocation of photosynthetic product (Brouwer, 1983), reducing AMF diversity following chemical fertilization (Johnson, 2010; Oehl et al., 2004). Consistent with previous studies, we found that chemical fertilizer application significantly decreased AMF alpha‐diversity which was negatively correlated with elevated soil nutrient level and crop yield, demonstrating that elevated soil nutrient level mediated by chemical fertilizer addition might decrease symbioses between plants and AMF (i.e., negative feedback; Lin et al., 2012).

However, a contrasting pattern of AMF diversity was found following organic fertilizer application relative to chemical fertilization, showing that elevated AMF diversity was positively correlated with increased soil nutrient level and crop yield, indicating that organic fertilizer addition might increase symbioses between plants and AMF. Previous studies have demonstrated that AMF could stimulate soil saprotrophs to decompose organic matter and increase soil nutrient levels, after which AMF rapidly transfer nutrients from decomposed organic matter to plants (Cheng et al., 2012; Xiang et al., 2015). Compared to chemical fertilizer, organic fertilizer needs to be transformed and degraded before being absorbed by plants. Plants may rely on AMF to facilitate the transformation process of organic fertilizer (Cheng et al., 2012), which may increase their allocation of belowground carbon to increase AMF diversity following organic fertilizer application. This finding suggests that there might be a positive feedback among soil nutrients, plants, and AMF following organic fertilizer addition.

Empirical studies also demonstrated that soil pH was a crucial factor in shaping soil AMF alpha‐diversity (Beenhouwer et al., 2015; Hazard et al., 2013; Kohout et al., 2015), which is consistent with our study (Table 1). A previous study showed that soil acidification significantly inhibited the growth of AMF and decreased AMF alpha‐diversity (Kohout et al., 2015). In this study, chemical fertilization, rather than organic fertilization, triggered soil acidification, which might further trigger the different patterns of AMF diversity following chemical and organic fertilization, in addition to fertilization‐type‐induced changes in symbioses between plants and their AMF partners.

## 5 CONCLUSIONS

In conclusion, our study has shown the differential responses of AMF community composition and diversity to chemical and organic fertilizer additions. We suggest the following pattern for long‐term fertilization in agricultural ecosystem: Chemical fertilization decreases the dependence of the plants on their AMF partners (Lin et al., 2012) and simultaneously decreases soil pH to inhibit AMF growth (Kohout et al., 2015), which triggers negative feedback between plants and AMF (Johnson, 1993). However, organic fertilization strengthens symbiotic relationships and shows positive feedback between plants and AMF. Our study demonstrated that application of organic manure might be superior to chemical fertilizers in sustainable agriculture. However, there are certain restrictions in application of organic fertilizers in some areas with less available organic materials. This work contributes to a more complete picture of the effect of long‐term fertilization on soil AMF community. However, our research cannot elucidate how the AMF community responds to fertilization over different timescales and soil depth gradients. Moreover, we did not explore the effect of fertilization on rhizospheric AMF symbionts. Lastly, we did not measure the overall AMF community abundance by quantitative real‐time PCR. Future work should clearly address these limitations to better predict how fertilization will affect agricultural ecosystem.

## CONFLICT OF INTERESTS

The authors declare no conflict of interest.

## AUTHOR CONTRIBUTIONS

XX and DL designed the experiment. JL, DL, and JZ completed the field sampling. JL and JZ performed data analysis and prepared figures. JL and XX wrote the manuscript. JZ, DL, and CX contributed to the revision of the manuscript.

## ETHICS STATEMENT

None required.

## Data Availability

The raw data were deposited in the European Nucleotide Archive (ENA, PRJEB30528).

## APPENDIX 1

**Table A1:** Summary of the main variables (soil properties and crop yield) of sampling sites

Variables	Control	NPK	2NPK	Manure	NPKM
Soil pH	5.24 (0.07)^c^	4.89 (0.11)^d^	4.82 (0.05)^d^	6.52 (0.06)^a^	6.12 (0.05)^b^
Total nitrogen (g/kg)	0.94 (0.03)^b^	0.95 (0.05)^b^	1.02 (0.07)^b^	1.24 (0.06)^a^	1.22 (0.09)^a^
Total phosphorus (g/kg)	0.60 (0.08)^c^	0.77 (0.12)^c^	0.98 (0.03)^b^	1.78 (0.12)^a^	1.94 (0.13)^a^
Soil organic carbon (g/kg)	6.81 (0.09)^d^	8.74 (0.27)^c^	9.03 (0.39)^c^	10.5 (0.44)^b^	11.2 (0.34)^a^
Soil NO_3_^−^ (mg/kg)	4.03 (0.33)^b^	5.37 (1.79)^ab^	4.89 (1.67)^b^	6.52 (0.37)^a^	6.92 (1.04)^a^
Soil NH_4_^+^ (mg/kg)	0.57 (0.15)^b^	0.81 (0.04)^ab^	1.01 (0.34)^a^	0.62 (0.18)^b^	0.72 (0.04)^ab^
Available phosphorus (mg/kg)	15.6 (2.82)^d^	28.1 (9.93)^d^	52.9 (4.42)^c^	225 (11.9)^b^	254 (5.5)^a^
Dissolved organic C (mg/kg)	28.1 (3.31)^d^	43.0 (2.10)^c^	44.9 (3.75)^c^	78.0 (2.63)^b^	88.6 (2.56)^a^
Dissolved organic N (mg/kg)	2.05 (0.43)^b^	2.33 (0.31)^b^	2.11 (0.11)^b^	5.95 (0.22)^a^	5.51 (0.17)^a^
Corn yield (kg/ha)	832 (67)^e^	3,705 (234)^d^	5,813 (484)^c^	7,013 (641)^b^	7,876 (225)^a^

Note

The values in brackets represent the standard deviation of the mean. Different letters behind brackets represent significant differences from Tukey HSD comparisons (*p* < .05).

**Table A2:** Differences in AMF community composition across the different treatments examined by analyses of similarities (ANOSIM)

Treatments	ANOSIM	
	*r*	*p*
Control versus (NPK + 2NPK)	**.556**	**.013**
Control versus (Manure + NPKM)	**.698**	**.007**
(NPK + 2NPK) versus (Manure + NPKM)	**.943**	**.005**

Significant correlations are shown in bold (*p* < 0.05).

**Table A3:** Variance partitioning analysis (VPA) showing the effects of variables on the soil AMF community composition performed in the vegan package of R project

Variables	Explained
	%
pH	9.42
TN	10.76
TP	–
NO_3_^−^	–
NH_4_^+^	–
AP	25.89
SOC	11.56
DOC	–
DON	–

Abbreviations: –, variables which have been excluded due to multicollinearity; AP, available phosphorus; DOC, dissolved organic carbon; DON, dissolved organic nitrogen; SOC, soil organic carbon; TN, total nitrogen; TP, total phosphorus.

**Table A4:** Pearson correlations (*r*) between the relative abundance of dominant AMF taxa and variables (soil properties and crop yield)

	*Glomerales*	*Paraglomerales*	*Archaeosporales*	*Diversisporales*
pH	**0.866** **	**−0.861** **	−0.377	−0.411
TN	**0.676** **	**−0.658** **	−0.366	−0.430
TP	**0.774** **	**−0.764** **	−0.328	−0.431
NO_3_^−^	0.471	−0.449	**−0.639** *	**−0.536** *
NH_4_^+^	−0.338	0.334	**0.522** *	0.336
AP	**0.825** **	**−0.817** **	−0.354	−0.423
SOC	**0.573** *	**−0.568** *	−0.201	−0.371
DOC	**0.767** **	**−0.760** **	−0.321	−0.440
DON	**0.847** **	**−0.839** **	−0.428	−0.487
CY	0.480	−0.471	−0.144	−0.297

Abbreviations: AP, available phosphorus; CY, corn yield; DOC, dissolved organic carbon; DON, dissolved organic nitrogen; SOC, soil organic carbon; TN, total nitrogen; TP, total phosphorus.

Significant correlations are shown in bold (*p* < 0.05).

* *p* < .05.

** *p* < .01.
